# Evaluating a process of academic detailing in primary care: an educational programme for acute kidney injury

**DOI:** 10.1186/s12909-019-1659-y

**Published:** 2019-07-09

**Authors:** Rebecca A. Noble, Joanna C. McKinnell, Sue Shaw, Sally Bassett, Lynn Woods, Mufaza Asrar, Nitin V. Kolhe, Nicholas M. Selby

**Affiliations:** 1 Department of Renal Medicine, Royal Derby Hospital, University Hospitals of Derby and Burton NHS Foundation Trust, Uttoxeter Road, Derby, DE22 3NE UK; 2 Southern Derbyshire Clinical Commissioning Group, Derby, UK; 3 Centre for Kidney Research and Innovation, Division of Medical Sciences and Graduate Entry Medicine, Royal Derby Hospital Campus, University of Nottingham, Derby, UK

**Keywords:** AKI, Primary care, Academic detailing, Peer-review, Nephrology

## Abstract

**Background:**

Primary care has a significant role in AKI management: two-thirds of AKI originates in the community. Through academic detailing (an evidence-based educational approach) we aimed to implement and measure the effect of a primary care-based education programme based around academic detailing and peer-reviewed audit.

**Methods:**

The education programme took place across a large clinical commissioning group (CCG) consisting of 55 primary care practices. All 55 practices participated in large group teaching sessions, 25 practices participated in academic detailing and 28 of the remaining 30 practices performed internal AKI audit. Over a 12 month period, an educational programme was delivered consisting of large group teaching sessions followed by either academic detailing sessions or self-directed AKI audit activity. Academic detailing sessions consisted of a short presentation by a consultant nephrologist followed by discussion of cases. Qualitative feedback was collected from all participants at peer review sessions. Web-based, CCG-wide questionnaires assessed baseline and post-intervention knowledge levels.

**Results:**

Nine hundred ninety-six individuals completed the questionnaires (556 at baseline, 440 at 1 yr., 288 participated in both). Exposure to AKI teaching, self-reported awareness and confidence levels were higher in the second questionnaire. There was a significant increase in the percentage of correct answers before and after the intervention (55.6 ± 21% versus 87.5 ± 20%, *p* < 0.001). Improvements were also seen in practices that did not participate in academic detailing. 92.9% of participants in the academic detailing sessions ranked their usefulness as high, but half of participants expressed some anxiety about discussion of cases in front of peers.

**Conclusion:**

Primary care education can improve knowledge and awareness of AKI. Small group teaching with involvement of a nephrologist was popular, although there were mixed responses to group discussion of real cases. Academic detailing did not appear more effective than other educational formats.

**Electronic supplementary material:**

The online version of this article (10.1186/s12909-019-1659-y) contains supplementary material, which is available to authorized users.

## Background

Acute Kidney Injury (AKI) is common, occurring in 5–10% of hospital admissions and is associated with poor patient outcomes, in particular substantial increases in mortality [1]. AKI also has long-term sequelae, notably an increased risk of Chronic Kidney Disease (CKD) [2]. Whilst AKI has traditionally been viewed as a remit of secondary care, this is changing. Approximately two-thirds of hospitalised patients have AKI on admission, inferring that prevention needs to encompass pre-hospital management [3]. AKI can occur and be managed in primary care, although variation in the provision of care may increase the risk of subsequent CKD in this setting [4]. Finally, medication changes during a hospital stay emphasise the importance of post-discharge AKI management that does not always occur [5].

There is therefore both need and benefit to increase awareness and knowledge levels of AKI in primary care. Amongst different educational formats, academic detailing is an approach that has gained popularity. Academic detailing involves experts visiting health care professionals in their own environment to provide tailored education on specific topics. A systematic review (including 69 studies and over 15,000 health care professionals) concluded that academic detailing can be effective at improving prescribing practices and in some situations, improves clinical practice [6] Conversely, there is a cost in terms of efficiency with multiple sessions required to reach a large audience. Academic detailing has been evaluated in a small number of studies in CKD, leading to improvements in clinical practice in motivated primary care groups [7] but to date has not been used in the setting of AKI. We therefore designed a model of academic detailing sessions coupled to peer review audit activities to deliver a primary care AKI educational programme across an entire clinical commissioning group (CCG). To do so, we linked the educational programme to local commissioning tools, and we measured its acceptability and effectiveness.

## Methods

This study used educational tools with the aim of improving knowledge and awareness of AKI in primary care healthcare providers, predominantly General Practitioners (GP), Advanced Nurse Practitioners (ANP) and Practice Nurses. Outcomes measured included feedback from attendees of academic detailing sessions, and knowledge tests before and after the educational intervention, carried out via an online questionnaire. The project was categorised as quality improvement (QI) and as such was not formally reviewed by a NHS Research Ethics Committee and we therefore did not require written consent from participants. However, all participants did give verbal consent to participate in the sessions as well as the questionnaires and feedback forms.

### Setting

The educational programme was rolled out across a single large Clinical Commissioning Group (Southern Derbyshire CCG) that encompasses 55 GP practices and provides primary care services to over 548,000 patients [8]. The programme was linked to the NHS England ‘Think Kidneys’ AKI Pathfinder project, which was established to explore how commissioning tools could be developed to improve management of AKI across primary and acute care in a range of care settings [9]. The education programme ran throughout financial year 2015–16, and participation of primary care practitioners was incentivised within a Locally Enhanced Service (LES) that required each practice to complete the online questionnaires and perform at least one AKI audit (which could be participation in an academic detailing session) to meet the criteria for reimbursement.

### Intervention

**Fig. 1:**
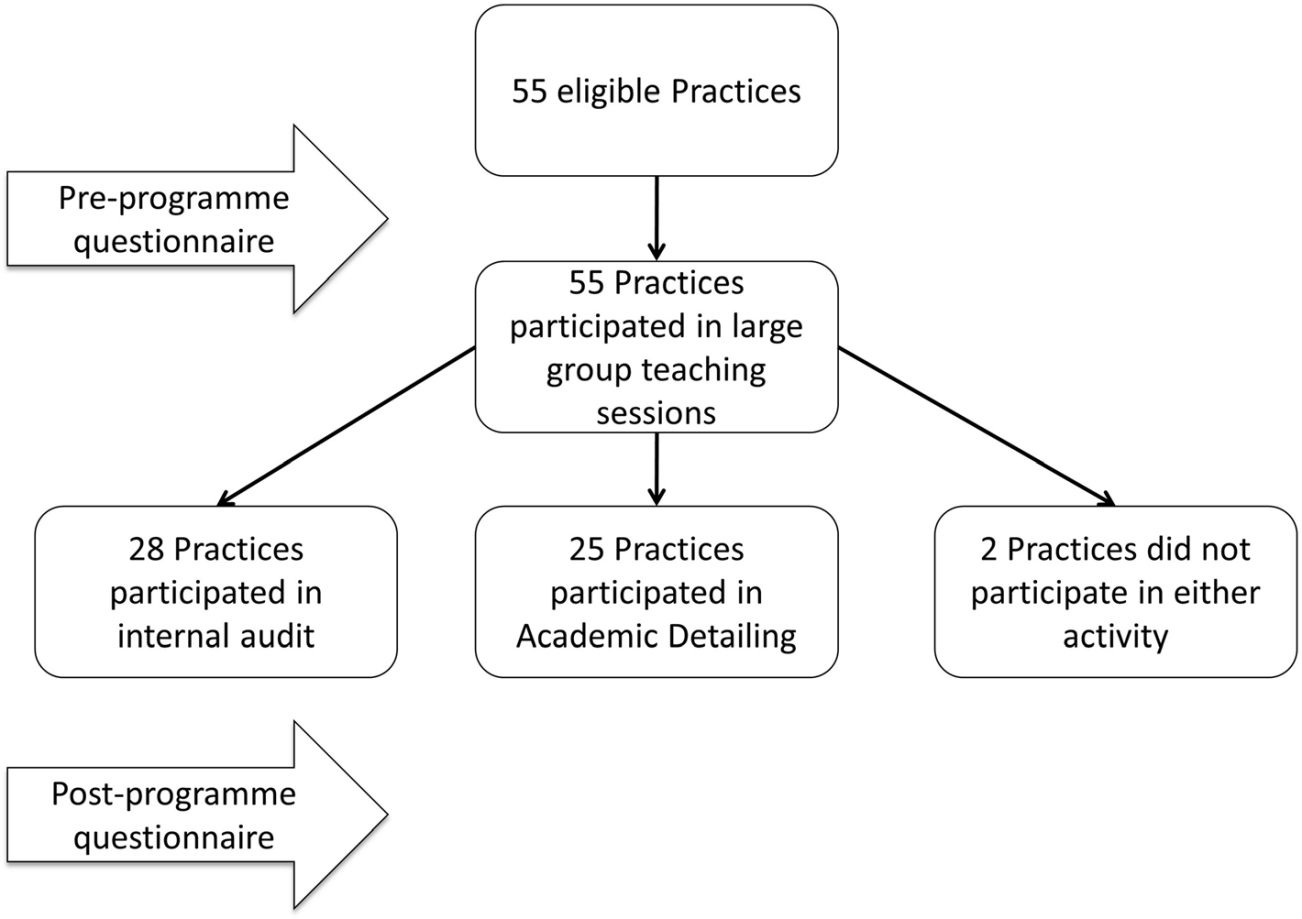
Flow chart demonstrating intervention

The educational programme was delivered between April 2015 and April 2016 and consisted of two components: large group teaching sessions and academic detailing sessions at individual GP practices (Fig. 1). The large group teaching consisted of nephrology and clinical biochemistry lectures at a primary care quality forum towards the beginning of the 12-month period at which all practices had at least one representative. Subsequently, a renal pharmacist delivered AKI teaching at a practice nurse forum. In parallel, all practices were offered academic detailing, where one of four specialists (three nephrologists, one GP with specialist AKI interest) delivered a standardised presentation to the practice. This was followed by practice-specific discussions of AKI cases with expert input. Practices were provided with a list of cases from their practice who had previously sustained AKI to allow opportunity for preparation prior to the session. All attendees at the peer review sessions were given graded evaluation forms. A web-based primary care AKI guideline was also launched [10].

Prior to delivering the programme an online questionnaire to assess knowledge levels and awareness was distributed to the 55 General Practices, with approximately 450 GPs [11], within Southern Derbyshire CCG. Following completion of the academic detailing programme, a follow-up knowledge questionnaire was distributed again to all practices between January and March 2016.

### Pre- and post-Programme questionnaire

Both pre- (2015) and post- (2016) programme questionnaires were completed using a Survey Monkey platform with participants asked to provide consent for analysis of anonymised aggregate data. The content of the two questionnaires was different, with a different number of knowledge questions to avoid repetition-recall bias. The knowledge questions were multiple-choice questions (MCQs) based on AKI cases, and although some questions were different they assessed the core themes of clinical knowledge of AKI diagnosis, investigation and management. These were written by consultant nephrologists and based on similar work targeting hospital clinicians [12]. In addition, there were some survey style questions in both questionnaires, which differed depending on whether the questionnaire was completed pre- or post- intervention. The 2015 survey contained questions about preferred types of education, whilst the 2016 survey focussed on self-reported awareness of AKI developments within the last year. Complete knowledge based questionnaires are included as Additional file 1. 

### Graded evaluation forms

Graded paper evaluation forms were completed by attendees at the academic detailing sessions. Participants were asked to rank the usefulness and novelty of various aspects of the session. There were also questions about preferred format of delivery and a space for free text feedback. The forms were completed anonymously to encourage honest responses, and submitted directly to the CCG for data extraction.

### Data analysis

Data from the pre- and post-programme questionnaires and the graded evaluation forms were analysed using SPSS v.22. Participants included General Practitioners (GPs), Advanced Nurse Practitioners (ANPs), Foundation Year Doctors, Practice Nurses, Pharmacists, and Healthcare Assistants. Analyses were performed for all participants combined, with subgroup analyses for GPs only and Nurses only (ANPs and Practice Nurses). Results are expressed as mean ± standard deviation (SD) and median (interquartile range, IQR) for parametric and non-parametric data respectively. Paired T-test (or non-parametric equivalent) were used to compare continuous variables and categorical data were compared using Chi-squared tests. The null hypothesis was accepted for *p*-values ≥0.05.

## Results

Nine hundred ninety-six individuals completed the online questionnaire. There were 556 respondents to the pre-programme survey, of whom 341 were GPs (76% of the GPs in Southern Derbyshire CCG). In the post-intervention questionnaire, there were 440 respondents, of whom 270 were GPs (60% of GPs in the CCG). Of the second cohort, 288 (66.4%) had completed the first survey. Overall, the distribution of healthcare professions was similar across both cohorts. In 2015, 61.3% were GPs, 27.2% were practice nurses and 7.2% were ANPs with the corresponding proportions for 2016 begin 61.5, 26.2 and 7.3% respectively. The remaining respondents comprised of pharmacists, healthcare assistants and junior doctors. All 55 practices within the CCG had at least one representative at the large group teaching sessions (part 1 of the intervention). For part 2 of the intervention, 25 practices participated in the academic detailing programme. Of the remaining 30 practices, 28 performed internal AKI audit without consultant nephrologist involvement.

### Knowledge-based questionnaire

There was a significant increase in self-reported confidence in making a diagnosis of AKI after the intervention as compared with baseline. 153 (27.5%) of respondents felt confident in making this diagnosis in 2015, increasing to 258 (59.2%) in 2016 (*p* < 0.001). There was also a significant increase in the respondents who had attended AKI teaching within the last 12 months 124 (22%) versus 282 (64%), *p* < 0.001), and in those who had read an AKI article in the last 12 months (326 (59%) versus 348 (79%), *p* < 0.001).

**Fig. 2:**
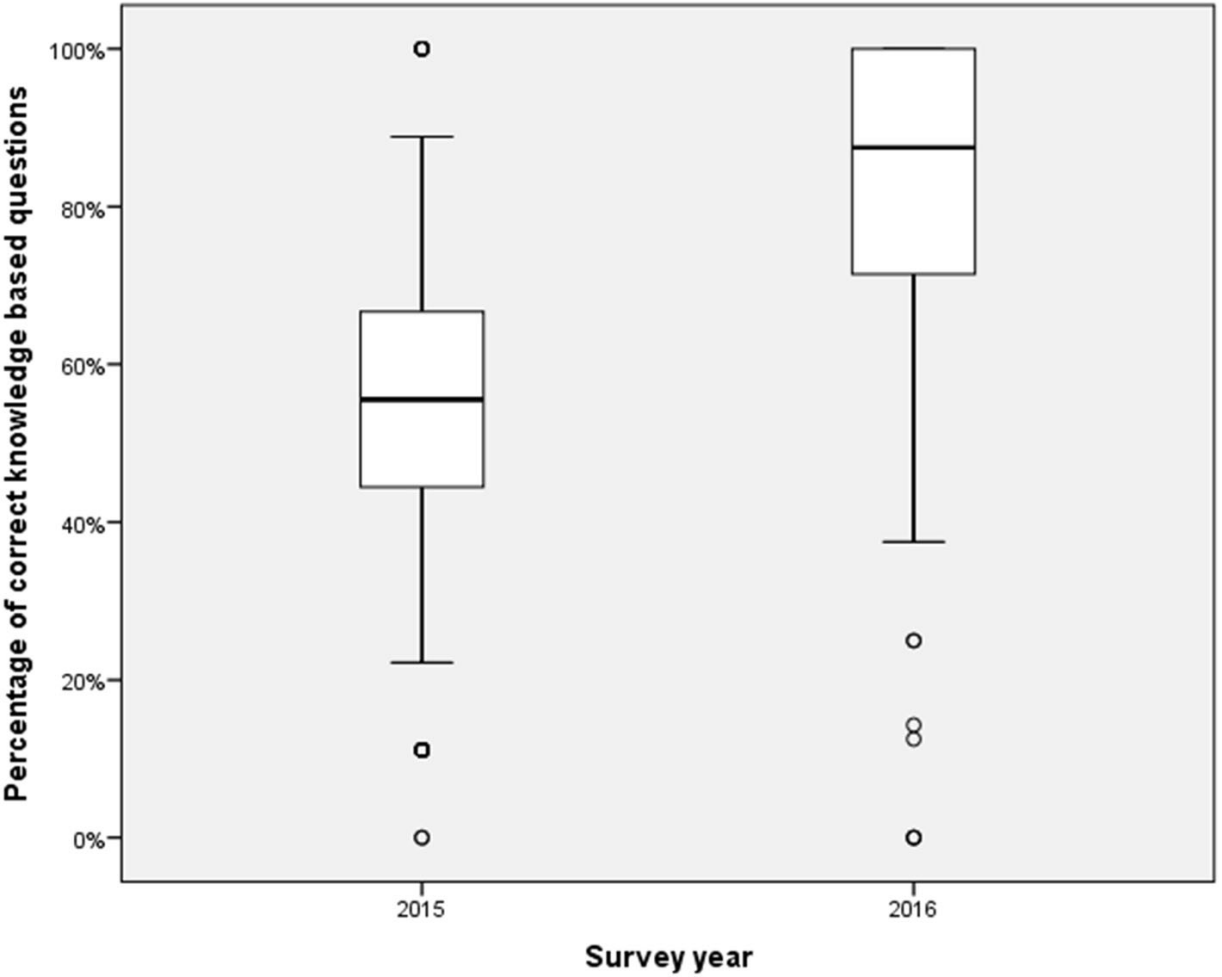
Percentage of correct knowledge based questions in 2015 (pre-intervention) and 2016 (post-intervention). For comparison between two groups, *p* < 0.001

In order to reduce the impact of repetition-recall bias, the knowledge and case-based management questions differed between the pre- and post-programme knowledge questionnaire (9 questions in 2015, 8 in 2016). There was a significant increase in the percentage of correct answers before and after the intervention (55.6 ± 21% versus 87.5 ± 20%, *p* < 0.001), shown in Fig. 2. Those questions that were directly comparable were analysed individually as follows: knowledge of AKI stages (question 6, 34.4% (*n* = 191) correct in 2015 vs 93.6% (*n* = 409) in 2016, *p* < 0.001); definition of stage 1 AKI (question 7, 41.2% (*n* = 229) correct in 2015 vs 88.9% (*n* = 375) in 2016, *p* < 0.001); and actions to reduce the risk of AKI (42.6% (*n* = 237) 2015 vs 92.7% (*n* = 403) 2016, *p* < 0.001). Similar patterns in improvement were seen when comparing the results only from those participants who took part in both questionnaires.

There were 451 respondents from practices that participated in academic detailing and 523 from those that did not, with a similar mix of respondents between groups (in non-participating practices 308 (58.9%) were GPs versus 249 (55%) in participating practices, *p* = 0.18). Exposure to AKI education and published articles increased by a similar magnitude in both groups (60.9 and 55.9% at baseline in non-participating and participating practices respectively, increasing to 80.4 and 78.4% post-intervention, *p* = 0.253 for comparisons between groups, *p* < 0.001 for comparisons between time points). Improvements in scores for knowledge based questions were also similar across groups: the percentage of correct answers was similar at baseline (55.8 ± 21% in non-participating practices versus 55.0 ± 21% in participating practices, *p* = 0.98) and increased post intervention to 83.2 ± 20% in non-participating practices versus 79.3 ± 20% in participating practices (*p* = 0.57 for comparison between groups, *p* < 0.001 for comparisons between time points).

The pattern for improvement in the group overall was also seen across the different roles within the multi-disciplinary team. For GPs (341 participants in 2015 survey, 270 in 2016), the mean score for the knowledge-based questions was 65.0 ± 16% at baseline improving to 87.7 ± 17% in the 2016 survey (*p* < 0.001). Similarly, ANPs and Practice nurses (191 in 2015, 147 in 2016) scored an average of 39.3 ± 19% at baseline improving to 71.7 ± 22% post intervention (*p* < 0.001). However, there was a difference between the two groups in self-reported confidence in diagnosing AKI. Both groups demonstrated improvements, but after the intervention only 20.3% of ANPs and Practice Nurses felt confident in making a diagnosis of AKI compared with 82.2% of GPs. As part of the baseline questionnaire, both GPs and ANPs/Practice Nurses felt peer group learning and face-to-face education would be useful approaches for primary care.

### Academic detailing evaluation

There were 115 responses to the graded evaluation forms from the 25 practices that participated in academic detailing sessions; 21 were excluded as they were from non-clinical staff, leaving 94 in the analysis (of whom 89.4% were prescribers). The spread of professions was: GPs 69%, Practice Nurses 12%, Advanced Nurse Practitioners 3%, Healthcare Assistants 4%, Foundation Doctors 2%, the remaining 10% did not specify their role.

**Fig. 3:**
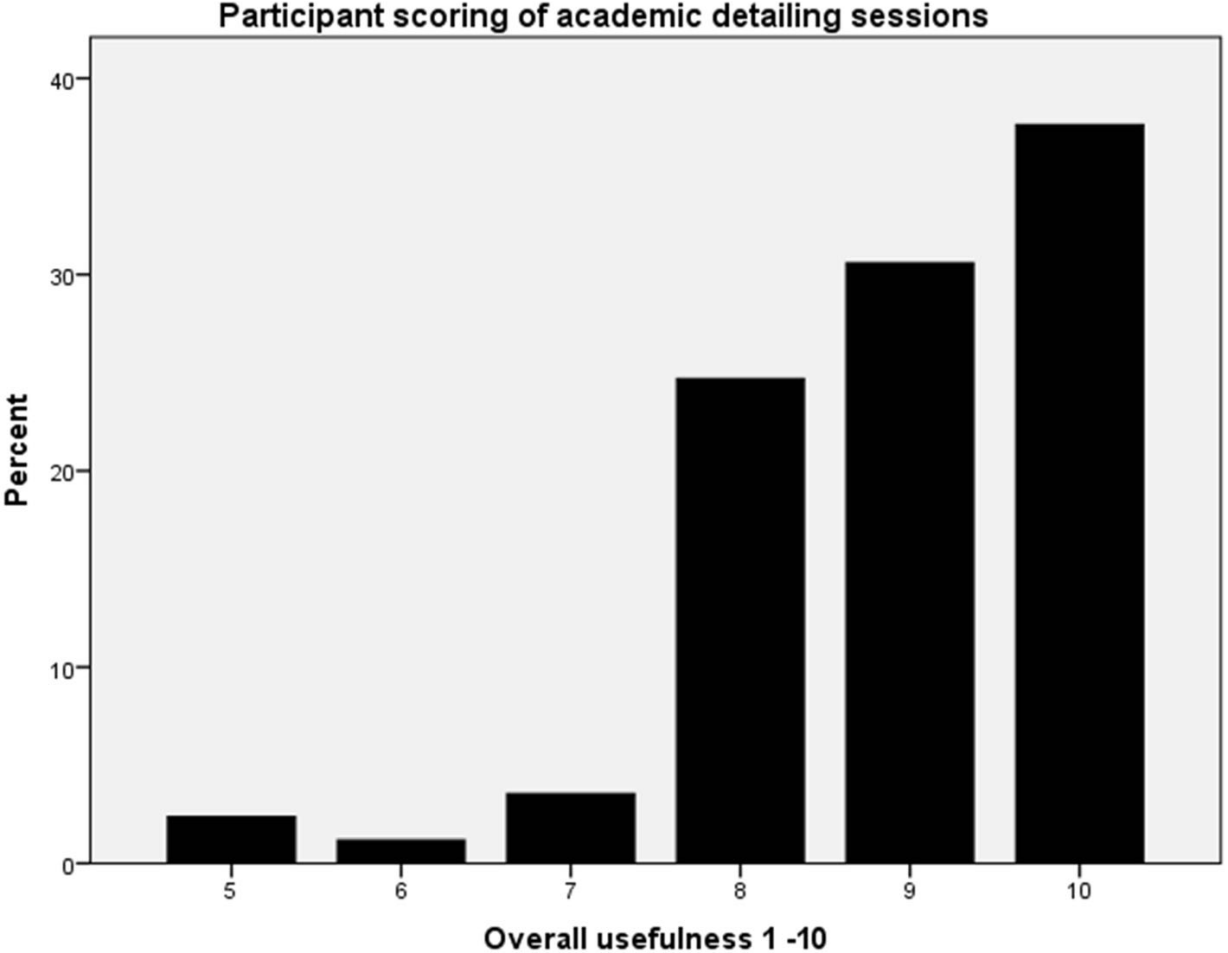
Overall usefulness of peer review session as reported by participants, ranked using a Likert scale (1–10) where 1 is low and 10 is high

Overall usefulness of the academic detailing session was ranked using a Likert scale, with 87 participants (92.9%) ranking usefulness as ≥8 (high). These data are shown in Fig. 3. Participants were asked to rank the perceived usefulness of different elements of the sessions, with the presence of a nephrologist being selected as the most useful aspect by 75.5% (*n* = 71). In other questions, 69.1% (*n* = 65) participants felt that discussions of AKI cases improved the sessions, quoting reasons such as increased relevance of the education and allowing practical lessons to be learnt. In line with the presence of a nephrologist being ranked the most useful aspect of the sessions, 88.3% (*n* = 83) felt the case-based discussions were improved by this presence. In terms of whether participants felt apprehensive about discussing cases in front of their peers, 47.9% said they were not at all apprehensive prior to the session (Likert score 1), 36.1% expressed some degree of apprehension and 16% ranked their apprehension as ≥6 (10 being significant apprehension). This was largely unchanged after the session.

Participants were also asked to rank the different topics within the sessions in terms of their perceived usefulness and how novel the information provided was. These data are shown in Table 1. ‘AKI management in primary care’ was scored as the most useful, with relatively small differences between the other topics. Topics that were ranked as more useful were not necessarily those that were perceived as the most novel – for example information about the NHS England AKI detection algorithm (AKI Warning Stage results) was scored as the most novel topic but was ranked low in terms of perceived usefulness of information. Two topics were ranked low in both categories: risk factors for AKI and sick day guidance.

**Table 1:** Ranking of topics by participants of academic detailing sessions by perceived usefulness of information (left) and by how novel the information was felt to be (right). Results shown are the topics placed in order of most highly ranked (1) to lowest ranked (7)

Ranking of topics by participants of academic detailing sessions in order of perceived usefulness of information	Ranking of topics by participants of academic detailing sessions in order of perceived novelty of information
1. Managing AKI in primary care	1. AKI warning stage issued as test result from biochemistry
2. AKI diagnostic criteria	2. Managing a patient after an episode of AKI
3. Implications of AKI for patient outcomes	3. AKI diagnostic criteria
4. Managing a patient after an episode of AKI	4. Implications of AKI for patient outcomes
5. Risk factors for AKI	5. Managing AKI in primary care
6. Sick day guidance and reducing risk of AKI	6. Sick day guidance and reducing risk of AKI
7. AKI warning stage issued as test result from biochemistry	7. Risk factors for AKI

83 (88.3%) participants reported that their practice regarding AKI would change as a result of the session and when asked for specific areas in which this would happen, a variety of answers were given including: prevention/monitoring in at risk patients (20.7%); application of sick day guidance (15.5%); improved post AKI follow up (12.1%); and better recognition of AKI in patients at risk (10.3%). Free text qualitative feedback was positive with examples given in Table 2. 88.3% stated they would want to participate in similar sessions in the future.

**Table 2:** Free text qualitative feedback from academic detailing evaluation sessions

Free text comments from participants of academic detailing sessions	
Mix of presentation, case note audit & informal discussion was good	
Liked the whole relaxed chat thing	
Brilliant	
Definitely a valuable learning experience. Enough information, down to earth, very practical and well delivered talk by Consultant	
Ensuring each participant bought a case would improve case discussions	
Excellent	
Excellent	
Excellent & helpful	
Excellent thanks	
Fantastic, Consultant had very good manner in presenting & was approachable	
Discussion of cases didn’t really happen and would have been useful	
Good session. Great to have some interaction between primary and secondary care	
Great evening, case discussions fell a bit flat as cases not prepared by GPs & some cases did not illustrate problems for GPs well. This form is quite long & complicated.	
Helpful to have an expert to field questions and highlight guidelines	

When asked to rate their preferences on educational style, 60.5% rated discussion of cases with specialist input as their preferred method of delivery, the second most popular education style was didactic large group presentations (43%) eLearning was not rated highly; this was mirrored in the results from the online questionnaire, where 98.7% of participants preferred face-to-face teaching as opposed to e-learning approaches.

## Discussion

We report the effects of a commissioning-supported educational programme that aimed to increase knowledge and awareness of AKI in primary care. Whilst the overall programme resulted in significant improvements in these areas, the approach of academic detailing and peer review audit did not appear to be more effective than other forms of education.

Academic detailing was originally described as an educational method to improve medication prescribing. It involves educational outreach, and is based on behavioural science principles including active participation in sessions, establishing credibility of the essential messages and providing positive reinforcement [13]. Almost 30 years ago, a randomised controlled trial of academic detailing demonstrated how personalised educational visits to office-based physicians reduced unnecessary prescribing and resulted in significant cost savings [14]. Similar results have been reported by others [6]. More recently, academic detailing has been used to educate health care workers on topics other than medication prescribing. The approach has been shown to be effective in improving clinical practice in CKD [7]. Academic detailing in tandem with performance feedback and practice facilitation resulted in improved uptake of CKD guidelines in primary care, leading to changes in process measures (improved prescribing and CKD monitoring) and motivated practices were able to recruit other practices to participate [7]. To our knowledge, academic detailing has not been used previously for AKI education. In this study, academic detailing consisted of a single visit to participating practices, with key educational messages based on local and national primary care AKI guidelines [9]. This was combined with peer review of previous AKI cases from each individual practice.

The educational programme was tested in combination with an NHS commissioning tool that incentivised participation (Locally Enhanced Service, LES). The commissioning approach was effective, resulting in a high number of respondents to the baseline and post-intervention questionnaires. There are approximately 450 GPs in Southern Derbyshire CCG [11], making the response rates 75% in 2015 and 60% in 2016. This is far higher than would be expected from a voluntary questionnaire and provides reassurance in terms of the representativeness of results. In addition, all but two practices participated in some form of AKI audit, although less than half chose to participate in the academic detailing sessions. Of those who did participate in academic detailing, preparation of cases was noted to be variable. We can speculate that without linking to a LES, it is likely that our educational programme would not have achieved the same degree of penetration across the entire CCG.

The aim of our intervention was to evaluate whether academic detailing could modify knowledge, in this instance in AKI. Our results demonstrated large increases in awareness and self-reported confidence levels in primary care practitioners following the educational intervention. Knowledge scores increased, demonstrating that the overall programme of education was successful, and importantly benefits were seen in both medical and nursing practitioners. However, the magnitude of improvement was the same in those that did and did not participate in academic detailing sessions. It is notable that participants in the 2016 online questionnaire reported significantly higher rates of exposure to both AKI education sessions and journal articles about AKI as compared to 2015, and at least one practitioner from every practice attended the large group lectures. Therefore, improvements in 2016 knowledge scores may in part have resulted from self-directed learning and benefitted from a rising awareness of AKI in general, in addition to that gained directly from the educational intervention. A repeat survey now, a year on may provide some insight here. It is also possible that there may be more subtle benefits from the academic detailing approach that were not detected by the methods or content of the online questionnaire. This would potentially be supported by the positive feedback from those who did participate (92.9% ranked the usefulness of the academic detailing session as high, and nephrology input was also rated highly). Free text qualitative feedback included comments such as “Great to have some interaction between primary and secondary care,” and “Helpful to have an expert to field questions and highlight guidelines”. However, there are significant resource implications to academic detailing, which requires a large number of small-group teaching sessions to be delivered by senior clinicians, each session taking an average of 3 h of clinician time. Without clear benefit of this approach, more efficient models that integrate the most highly ranked aspects from the academic detailing sessions would seem to be preferable for the purpose of AKI education. For example, respondents to the online questionnaire and participants in the academic detailing sessions both showed a clear preference for face to face education (as opposed to e-learning), so larger group teaching sessions which include case-based discussions and opportunities for interaction may be a more sustainable model.

A second consideration is the variable perceptions towards the peer reviewed case discussions that we observed. Whilst only 16% reported higher levels of apprehension, less than half said that they were completely comfortable with this concept. Interestingly, the reported levels of apprehension did not change significantly after participating in a session, suggesting that preconceptions about this approach may not alter quickly. Coupled to this, less than half of the practices in the CCG chose to participate in academic detailing. A weakness of our study is that we did not collect data as to why practices chose not to participate, although we can speculate about possible reasons. These may include apprehension at the thought of discussing cases in front of their peers and workload. It may be that some practices simply preferred to undertake their own AKI audit activities.

Qualitative results from the peer review audit sessions showed participants felt the topics covered were useful, suggesting that the sessions were relevant, pitched at the right level and that AKI education does have a place in primary care. The novelty of different topics varied more across topics. This may provide some insights into the planning of future sessions, allowing prioritisation or de-prioritisation of topics depending on their familiarity. For example, sick-day guidance for AKI has been widely publicised in primary care in the UK, and this topic was one of those rated least novel and least useful. It was also important to note that the more novel topics were more likely to be cited as areas in which practice would change, for example improved recognition and follow up of patients with AKI.

There are some weaknesses to this study. Most importantly, the time-series design means that we cannot exclude that improvements in AKI awareness and knowledge levels changed over the time course of the study period independently of the intervention. However, the magnitude of improvement that we saw would suggest that this is unlikely to explain all of the results observed. Secondly, the study was not randomised which limits the conclusions we can draw when comparing practices that did and did not choose to participate in academic detailing, and we cannot exclude potential selection bias for example. We did not collect qualitative data from practices who did not participate in academic detailing; this may have generated additional insights. Thirdly, we did not collect data on clinical outcomes after the intervention i.e. whether increased awareness and knowledge led to a reduction in hospital admissions and improved follow-up. In a project of this size, we didn’t intend to measure effect on patient outcomes. Finally, we did not evaluate how the effect of the intervention may have differed if introduced without a commissioning framework.

## Conclusion

In conclusion, a CCG-wide education process resulted in significant improvements in AKI awareness and knowledge levels. Combining the education programme with a commissioning approach was effective, resulting in excellent levels of engagement. However, academic detailing does not appear to offer significant advantages over other forms of educational activity and more efficient models are recommended for the purpose of AKI education in primary care.

## Additional file

Additional file 1: Pre- and Post-Programme Questionnaires. Questionnaires used pre- and post-programme to assess knowledge as well as survey style questions. (DOCX 21 kb)

## Data Availability

The datasets generated and analysed during the current study are not publicly available as the project fell under the remit of a quality improvement project and therefore we do not have consent from participants to share data. Data may be made available from the corresponding author on reasonable request.

## Abbreviations

AKI: Acute Kidney Injury
ANP: Advanced Nurse Practitioner
CCG: Clinical Commissioning Group
CKD: Chronic Kidney Disease
GP: General Practitioner
IQR: Interquartile Range
LES: Locally Enhanced Service
MCQs: Multiple-choice questions
QI: Quality Improvement
SD: Standard deviation
