# Supplementary material for: Evaluating a process of academic detailing in primary care: an educational programme for acute kidney injury
Source: BMC Med Educ. 2019 Jul 9;19:253. doi: 10.1186/s12909-019-1659-y (PMC6617939; doi:10.1186/s12909-019-1659-y)
Supplement: Supplementary file 1 — Pre- and Post-Programme Questionnaires. Questionnaires used pre- and post-programme to assess knowledge as well as survey style questions. (DOCX 21 kb) [file 12909_2019_1659_MOESM1_ESM.docx]

**Pre-Programme Questionnaire (2015)**

1. Have you read an AKI article in last 12 months?

2. Have you attended AKI teaching in last 12 months?

3. Do you find face-to-face teaching useful?

4. Do you find e-learning useful?

5. Do you find peer group learning useful?

6. Are you interested in having AKI resources provided?

7. Are you confident in making a diagnosis of AKI?

**Knowledge questions:**

8. The 3 main causes of AKI are: (tick the answers you agree with)

- Autoimmune diseases
- Hypovolaemia
- Diabetes
- Sepsis
- Medication
- Hypertension
- Unsure

9. AKI is rare and should only be looked after by specialist kidney doctors. True/False

10. Most cases of AKI are hospital acquired. True/False

11. How many stages of AKI are there?

- 3
- 4
- 5
- Unsure

12. What is the correct definition of AKI stage 1?

- An increase of more than 3-fold from baseline
- An increase of 2-3 fold from baseline
- An increase of 1.5-2 fold from baseline or an increase of ≥27µmol/l (within a 48hr period)

13. Is anuria enough to diagnose AKI? Yes/No

14. In which of the following situations is eGFR a reliable estimate of kidney function?

- AKI
- Patient with a lower limb amputation
- Patient with a single kidney
- Malnutrition
- Pregnancy
- None of the above
- Unsure

**Case-based questions:**

15. A 75 year old lady comes to see you at your Practice having felt generally unwell for the last few days. You do some tests. Which of the following would most reliably indicate AKI?

- Haematuria and proteinuria on urinalysis
- Low Haemoglobin
- High PO4 or PTH level
- Doubling of serum creatinine over a period of one week
- Unsure

16. The blood test results: Na 140micromol/l, K 5.5micromol/l, creatinine 185micromol/l. Urinalysis does not show any abnormalities and MSU does not show the patient has a urinary tract infection. Medicines: Ramipril 10mg od, Furosemide 40mg od, Aspirin 75mgs od. The patient’s serum creatinine levels have been stable over the last 6 months with creatinine ranging from 94-104micromol/l. The patient rings the next day to say she is feeling more unwell, is vomiting but is still able to take fluid orally. Which ONE of the following is the most appropriate course of action to prevent possible AKI?

- See her and arrange immediate hospital admission
- See her and stop frusemide permanently
- See her and prescribe and anti-emetic to relieve vomiting
- See her and temporary stop Ramipril (for up to 48hrs)
- Unsure

**Post-Programme Questionnaire (2016)**

1. Have you read AKI article in last 12 months?

2. Have you attended AKI teaching in last 12 months?

3. Are you aware that from April 2016, AKI Warning Stage alerts will be generated on creatinine blood test U/E samples from primary care in which there has been a significant change from that patient’s usual value?

- Yes
- No
- Not sure

4. Is your practice issuing AKI sick day guidance as per the Prescribing Quality Scheme?

- Yes
- No
- Not sure

5. Are you confident in making a diagnosis of AKI?

- Yes
- No
- Not sure

**Knowledge questions:**

6. Select the three most important risk factors for developing AKI (please tick the answers you agree with):

- Inflammatory Bowel Disease
- Pemphigoid
- CKD
- Chronic Asthma
- Increasing age (being ‘Elderly’)
- Hypertension
- Having had AKI previously

7. All cases of AKI should be admitted to hospital? True/False

8. CKD (chronic kidney disease) occurs in a significant proportion of patients following an episode of AKI. True/False

9. Which stage of AKI is the most severe?

- 1
- 2
- 3

10. AKI is determined by a rise in serum creatinine with respect to an individual’s normal (baseline) value. Which of the following is the correct definition of AKI Stage 1?

- An increase of more than 3-fold from baseline
- An increase of 2-3 fold from baseline
- An increase of 1.5-2 fold from baseline or an increase of ≥27µmol/l (within a 48hr period)

**Case-based questions:**

11. A 75-year-old diabetic lady with CKD stage 3 comes to see you at your practice having had diarrhoea and vomiting for the last few days. She is taking Ramipril 10mg od, Nifedipine 30 mg od, Aspirin, 75mgs od, Gliclazide 40mg od. What specific advice would you give to reduce the risk of AKI?

- Stop all medications
- Prescribe anti-emetic and immodium
- As she is unwell, assume a urinary tract infection and commence empirical antibiotics
- Suspend ramipril for 48hrs, encourage oral rehydration and arrange early follow-up if not improving
- Unsure

12. One of your patients with a background of heart failure is discharged after an episode of AKI stage 3 associated with hypovolaemia. Prior to admission, her serum creatinine was 98μmol/l. At discharge, her creatinine is 143μmol/l. During the admission, aspirin, simvastatin and ramipril were all stopped. You restart her aspirin and simvastatin at her first follow up visit; what do you do regarding the ramipril?

- Avoid ramipril now she has had AKI, even though it is indicated for heart failure
- Restart ramipril at the same time as aspirin and simvastatin
- Refer to a cardiologist for advice
- Recheck her renal function, and restart ramipril when renal function has stabilised (even if worse than previously)
- Unsure

13. One of your patients with a background of heart failure is discharged after an episode of AKI stage 3 associated with hypovolaemia. Prior to admission, her serum creatinine was 98μmol/l. At discharge, her creatinine is 143μmol/l. During the admission, aspirin, simvastatin and ramipril were all stopped. You restart her aspirin and simvastatin at her first follow up visit; what do you do regarding the ramipril?

- Assess whether this really is AKI by looking at previous creatinine results and interpreting within the clinical picture
- Admit the patient to hospital
- Check medications
- Call renal team for advice
- Unsure
